# Smartphone addiction may be associated with adolescent hypertension: a cross-sectional study among junior school students in China

**DOI:** 10.1186/s12887-019-1699-9

**Published:** 2019-09-04

**Authors:** Yunfei Zou, Ning Xia, Yunqing Zou, Zhen Chen, Yufeng Wen

**Affiliations:** 1 School of Public Health, Wannan Medical College, No.22 Wenchangxi Road, Yijiang district, Wuhu City, 241002 Anhui Province China; 2 Industrial and Commercial College, Anhui University of Technology, No.8 Huang Chi Road, Gushu Town, Dangtu County, Ma’anshan City, 243100 Anhui Province China

**Keywords:** Adolescent hypertension, Body mass index, Obesity, Smartphone addiction, Sleep quality

## Abstract

**Background:**

Hypertension in children and adolescents is on the rise worldwide, especially in China. The prevalence of hypertension is related to many factors, such as obesity. In the era of smart phones, it is important to study the negative health effects of mobile phones on blood pressure. The purpose of this study was to investigate the prevalence of hypertension and its association with smartphone addiction among junior school students in China.

**Methods:**

A school-based cross-sectional study was conducted, including total 2639 junior school students (1218 boys and 1421 girls), aged 12–15 years old (13.18 ± 0.93 years), enrolled in the study by random cluster sampling. Height, weight, systolic blood pressure (SBP) and diastolic blood pressure (DBP) were measured following standard protocols, and the body mass index (BMI) was calculated. Overweight/obesity and hypertension were defined according to sex- and age-specific Chinese children reference data. The Smartphone Addiction Scale short version (SAS-SV) and the Pittsburgh Sleep Quality Index (PSQI) were used to assess smartphone addiction and sleep quality among the students, respectively. Multivariate logistic regression models were used to seek associations between smartphone addiction and hypertension.

**Results:**

The prevalence of hypertension and smartphone addiction among participants were 16.2% (13.1% for females and 18.9% for males) and 22.8% (22.3% for females and 23.2% for males), respectively. Obesity (OR = 4.028, 95% CI: 2.829–5.735), poor sleep quality (OR = 4.243, 95% CI: 2.429–7.411), smartphone addiction (OR = 2.205, 95% CI: 1.273–3.820) were significantly and independently associated with hypertension.

**Conclusions:**

Among the junior school students surveyed in China, the prevalence of hypertension was high, which was related to obesity, poor sleep quality and smartphone addiction. These results suggested that smartphone addiction may be a new risk factor for high blood pressure in adolescents.

## Background

It is well known that hypertension, or high blood pressure, is a major public health issue, also is one of the primary risk factors for cardiovascular diseases, including cerebrovascular stroke, atherosclerosis [1–3]. In China, hypertension is one of the most important causes of cardiovascular disease mortality, the latest figures (from the recent large-scale prospective China Kadoorie Biobank Study of 500,223 adults aged 35 to 74 years, in 10 diverse regions of China) suggest that 32.5% of participants (33.7% of men and 31.9% of women) had hypertension [4].

From a public health point of view, the prevention and control of hypertension are essential to maintain and promote human health, particularly in childhood, because many studies have provided ample evidence that hypertension in adults has its onset in childhood, that is, children and adolescents with elevated blood pressure (BP) could ultimately become recognizable hypertension in adults. Childhood BP is associated with BP in later life [5, 6]. Based on these observations, many scholars have pointed out that decreasing the level of BP in children and adolescents could help reduce the risk of elevated BP–related diseases in both childhood and adulthood [7]. In recent decades, many epidemiological studies have reported that overweight and obesity have a significant positive association with high blood pressure in pediatric populations [6, 8]. Treating obesity may be essential to preventing prehypertension and/or hypertension [9]. In recent years, the use of smart phones has become more and more popular in Chinese adolescents. Smartphone has numerous benefits for social and medical purposes. According to Research Report on Internet behavior of Chinese adolescents in 2014, by December 2014, the size of China’s youth Internet users had reached 277 million, accounting for 79.6% of China’s youth population. 87.6% of Internet users are teenagers under the age of 18 and who use smartphones to access the Internet [10]. The overall prevalence of Internet Addiction was 26.50% among adolescents in China [11], which is much higher than other Asian countries (range: 6.2–21.2%) [12, 13]. Internet addiction is associated with psychiatric disorders in adolescents including depression, anxiety, attention deficit and hyperactivity disorder and alcohol misuse [14]. Adolescents suffering from Internet addiction often avoid socialization with other people [15]. Internet addiction was found to affect sleep quality [16] and lead to negative impact on health-related quality of life [13] in adolescents. In contrast, less research was conducted to study the effects of Smartphone addiction on adolescents.

Smartphone can provide a variety of functions such as phone, camera, multimedia player, internet browser, navigation system, and e-mail service as well as facilitate social networking and game playing. Because of these powerful and attractive advantages, many adolescents overuse mobile phones, resulting in similar addiction symptoms [17].

Smartphone addiction is also called “mobile phone dependence”, “compulsive mobile phone overuse” or “mobile phone overuse”. These terms mainly describe the phenomenon of problematic mobile phone use. “Smartphone addiction” is the term typically used in the literature [18]. Smartphone addiction has been found to be an emerging public health problem.

The prevalence of hypertension among young people in China is on the rise. Many factors affect the occurrence of hypertension. The relationship between psychosocial factors and blood pressure has attracted more and more attention. Some studies have shown that smartphone addiction is associated with anxiety, depression and sleep disorders among adolescents [19]. To a certain extent, these mental disorders also affect blood pressure. But until now, there are few reports about the prevalence of hypertension among young people with mobile phone addiction. It is necessary to explore the role of smartphone addiction in the pathogenesis of hypertension in adolescents.

The aim of this study was to investigate the risk factors associated with hypertension in Chinese adolescents, especially the relationship with smartphone addiction.

## Methods

### Participants and procedures

A cross-sectional survey was performed during September and December 2014. A total of 2639 junior school students (1218 boys and 1421 girls) in grades 7 to 9, aged 12–15 years old (13.18 ± 0.93 years), were enrolled in this study by random cluster sampling. In our study, 1 district was randomly selected from 4 districts of Wuhu city, which is an important economic development center city of the Yangtze River Basin in China, and then randomly selected 4 middle schools in the area as the research subjects, of the selected schools, two classes in each grade were selected, and all students of the selected classes were invited to participate the survey. The study design and procedure was approved by the ethics committee of Wannan Medical College, Wuhu, China, and informed consent was obtained from all participants and their guardians before the survey.

### Measurements

All subjects had a thorough medical examination before the measurements were taken. They were free from cardiovascular or kidney disease and were not taking sleep, cardiovascular, or psychiatric medications. All measurements were conducted by a team of trained senior medical students in a standardized way.

Weight status was defined with body mass index (BMI, measured as weight in kilograms divided by height in meters squared). Obesity was defined as a BMI at or above the 95th percentile of the Working Group on Obesity in China (WGOC) definitions for overweight and obesity, overweight was defined as a BMI between the 85th and 95th percentiles [20, 21].

Height (cm) and weight (kg) were measured using the same type of apparatus and followed the standard protocols. Height was measured using metal column height-measuring stands to the nearest 0.5 cm with the participant’s no shoes, heels together, and head touching the ruler with line of aligned horizontally. Weight was measured, using lever scales to the nearest 0.1 kg with the participant’s bare foot and wearing a light cloth.

Systolic blood pressure (SBP) and diastolic blood pressure (DBP) were measured in the sitting position by a mercury sphygmomanometer after each subject had rested for at least 5 min with the appropriate cuff using the standard protocol in a quiet room. Three readings were recorded at 5-min intervals with complete deflation of the cuff between each reading. The average of the three readings was calculated.

DBP was defined via Korotkoff Sound 4 [22]. The reference values of SBP and DBP percentiles for Chinese children and adolescents were applied in this survey [23]. In this study, hypertension was defined as SBP and/or DBP above the 95th percentile for age and gender.

Smartphone addiction was measured using the Smartphone Addiction Scale short version (SAS-SV) [24]. The SAS-SV is a validated scale that contains 10 items rated on a dimensional scale (1 “strongly disagree” to 6 “strongly agree”). The total score ranges from 10 to 60, with the highest score representing the maximum presence of “smartphone addiction” in the past year. The original SAS-SV showed content and concurrent validity and internal consistency (Cronbach’s alpha: 0.91). Smartphone addiction cut-off values of ≥31 and ≥ 33 for male and female participants, respectively, were applied as suggested by Kwon et al. [24]. We modified the 8th item of the original SAS-SV “Constantly checking my smartphone so as not to miss conversations between other people on Twitter or Facebook” by replacing “Twitter or Facebook” with “WeChat, QQ, or micro-blog” so as to be more relevant to our study population. We invited English teachers to help translate the English version of SAS-SV into Chinese. The Chinese version of SAS-SV is internally consistent (Cronbach’s α = 0.844) [25].

Sleep quality was measured by using the Pittsburgh Sleep Quality Index (PSQI) [26], which is a widely used 19-item self-report questionnaire that evaluates subjective sleep quality over the past month. The 19 items yield seven component scores: sleep quality, sleep latency, sleep duration, sleep efficiency, sleep disturbances, use of sleep medication and daytime dysfunction. The sum of scores for these seven components yields the PSQI global score, which range from 0 to 21. Higher scores indicate poor sleep quality during the previous month. PSQI global scores > 7 were defined as “poor sleep quality”, as has been demonstrated in the Chinese population [27].

All questionnaires were in the self-report format. The basic sociodemographic data was collected in this survey, such as gender, age, grade.

### Data analysis

Descriptive results of categorical variables are described as frequency (%), and continuous variables are described as mean ± standard deviation (SD). Group differences were evaluated by Pearson’s chi-square test or t tests for category variable and continuous variable, respectively. The logistic regression model was performed to explore the factors associated with adolescent hypertension. P-values of < 0.05 were considered statistically significant. All data were analyzed using the SPSS version 20.0 (IBM Corporation, New York, USA). To avoid typing errors, we entered information into all questionnaires using a double-entry strategy in EpiData, version 3.1 (EpiData Association, Odense, Funen, Denmark).

## Results

### Participant characteristics

Of the 2639 participants, as shown in Tables 1, 1421 (53.8%) were males with a mean age of 13.22 years (SD = 0.95), and 1218 (46.2%) were females with a mean age of 13.12 years (SD = 0.90). The prevalence of poor sleep quality and smartphone addiction was 19.6% (19.0% for females and 20.1% for males), 22.8% (22.3% for females and 23.2% for males), respectively. None of this was statistically significant. Compared to the females, the males had a significantly higher age, height, weight, BMI, and SBP, but there was no obvious gender difference in DBP (see Table 1).

**Table 1:** Characteristics of the study population

	Total (*N* = 2639)	Female (*n* = 1218)	Male (*n* = 1421)	*P* value
Age (years)	13.18 ± 0.93	13.12 ± 0.90	13.22 ± 0.95	0.005
Grade				0.769
Grade 7	1005 (38.1)	472 (38.8)	533 (37.5)	
Grade 8	727 (27.5)	335 (27.5)	392 (27.6)	
Grade 9	907 (34.4)	411 (33.7)	496 (34.9)	
Height, cm	164.52 ± 8.51	161.25 ± 6.39	167.32 ± 9.08	< 0.001
Weight, kg	55.32 ± 11.64	52.01 ± 9.31	58.16 ± 12.65	< 0.001
BMI, kg/m^2^	20.35 ± 3.48	19.96 ± 3.15	20.68 ± 3.70	< 0.001
SBP, mmHg	108.08 ± 12.41	104.71 ± 11.08	110.97 ± 12.75	< 0.001
DBP, mmHg	65.64 ± 8.35	65.45 ± 8.04	65.80 ± 8.60	0.277
Sleep quality				0.487
Good	2121 (80.4)	986 (81.0)	1135 (79.9)	
Pool	518 (19.6)	232 (19.0)	286 (20.1)	
Smartphone addiction				0.586
Negative	2037 (77.2)	946 (77.7)	1091 (76.8)	
Positive	602 (22.8)	272 (22.3)	330 (23.2)	

Values are presented as mean ± SD or number (percentage) when appropriate

### Univariate analysis of the factors associated with smartphone addiction in adolescents

There was obvious difference in the rate of smartphone addiction among students of different grades. With the increase of body weight, addiction rate was also on the rise. Of the 518 students with poor sleep quality, 98.1% were addicted to smartphones. 264 of the 428 hypertensive students reached the standard of smartphone addiction (see Table 2).

**Table 2:** Univariate analysis of the factors associated with smartphone addiction in adolescents [n (%)]

	Total (*N* = 2639)	Smartphone addiction Positive (*n* = 602)	Smartphone addiction Negative (*n* = 2037)	*P* value
Grade				< 0.001
7	1005 (38.1)	166 (16.5)	839 (83.5)	
8	727 (27.5)	178 (24.5)	549 (75.5)	
9	907 (34.4)	258 (28.4)	649 (71.6)	
Gender				0.586
female	1218 (46.2)	272 (22.3)	946 (77.7)	
male	1421 (53.8)	330 (23.2)	1091 (76.8)	
BMI Percentile				< 0.001
< 85% (Normal)	1953 (74.0)	342 (17.5)	1611 (82.5)	
85 to 95% (Overweight)	472 (17.9)	163 (34.5)	309 (65.5)	
> 95% (Obesity)	214 (8.1)	97 (45.3)	117 (54.7)	
Sleep quality				< 0.001
Good	2121 (80.4)	94 (4.4)	2027 (95.6)	
Pool	518 (19.6)	508 (98.1)	10 (1.9)	
Hypertension				< 0.001
No	2211 (83.8)	338 (15.3)	1873 (84.7)	
Yes	428 (16.2)	264 (61.7)	164 (38.3)	

### Univariate analysis of the factors associated with adolescent hypertension

As shown in Table 3, Grades, gender, BMI level, sleep quality and smartphone addiction were all associated with hypertension in adolescents. Among them, the overall prevalence of hypertension in junior school students was 16.2% (13.1% for females and 18.9% for males). With the increase of grades, the prevalence of hypertension was on the rise. The prevalence of hypertension in normal weight, overweight and obesity groups were 11.4, 23.1 and 45.3%, respectively. Poor sleep quality and addiction to smartphone were associated with higher risk of hypertension.

**Table 3:** Univariate analysis of the factors associated with adolescent hypertension [n (%)]

	Total (*N* = 2639)	Hypertension group (*n* = 428)	Non-hypertension group (*n* = 2211)	*P* value
Grade				0.006
7	1005 (38.1)	134 (13.3)	871 (86.7)	
8	727 (27.5)	128 (17.6)	599 (82.4)	
9	907 (34.4)	166 (18.3)	741 (81.7)	
Gender				< 0.001
Female	1218 (46.2)	160 (13.1)	1058 (86.9)	
Male	1421 (53.8)	268 (18.9)	1153 (81.1)	
BMI Percentile				< 0.001
< 85% (Normal)	1953 (74.0)	222 (11.4)	1731 (88.6)	
85 to 95% (Overweight)	472 (17.9)	109 (23.1)	363 (76.9)	
> 95% (Obesity)	214 (8.1)	97 (45.3)	117 (54.7)	
Sleep quality				< 0.001
Good	2121 (80.4)	176 (8.3)	1945 (91.7)	
Pool	518 (19.6)	252 (48.6)	266 (51.4)	
Smartphone addiction				< 0.001
Negative	2037 (77.2)	164 (8.1)	1873 (91.9)	
Positive	602 (22.8)	264 (43.9)	338 (56.1)	

### Multivariate logistic analysis of factors associated with adolescent hypertension

Table 4 showed that gender, grade, BMI percentiles, sleep quality and smartphone addiction entered the multivariable logistic regression model. Compared with the control group, obesity (OR = 4.028, 95% CI:2.829–5.735), poor sleep quality (OR = 4.243, 95% CI: 2.429–7.411), smartphone addiction (OR = 2.205, 95% CI: 1.273–3.820) had a higher risk of hypertension.

**Table 4:** Multivariate logistic analysis of factors associated with adolescent hypertension

Variables	B	S.E	OR (95% CI)	*P* value
Gender	0.389	0.125	1.475 (1.156–1.883)	0.002
Grade				
7	reference			
8	0.351	0.156	1.420 (1.046–1.928)	0.025
9	0.351	0.146	1.420 (1.066–1.893)	0.017
BMI Percentile				
< 85% (Normal)	reference			
85 to 95% (Overweight)	0.35	0.148	1.419 (1.061–1.898)	0.018
> 95% (Obesity)	1.393	0.18	4.028 (2.829–5.735)	< 0.001
Sleep quality	1.445	0.285	4.243 (2.429–7.411)	< 0.001
Smartphone addiction	0.791	0.28	2.205 (1.273–3.820)	0.005

## Discussion

The main findings of this study were that the prevalence of hypertension in Chinese junior middle school students was high, and the risk factors include gender, grade, weight, sleep quality and smartphone addiction. In particular, this study found that smartphone addiction may be an independent and important predictor of hypertension in adolescents aged 12 to 15 years.

In the current study, the reference values of SBP and DBP percentiles for Chinese children and adolescents were used, which was established from eleven large scale cross-sectional BP surveys in mainland China from 2001 to 2010, covering four municipalities and seven provinces [23]. Our study revealed that overall HTN prevalence was 16.2% (13.1% for females and 18.9% for males), which was higher than that of a study conducted in Changsha [28], China (3.1% for HTN), while lower than in Shandong, China (23.3% for HTN) [29]. Another study conducted in the United Arab Emirates showed that the overall prevalence of HTN was 15.4% for boys, 17.8% for girls, respectively [30]. Bo et al. reported that, in 1999–2012, the prevalence of pre-HTN and HTN in US children and adolescents were 8.0 and 1.6%, respectively [31]. The reasons for these differences may be due to the difference in HTN criteria used, definition of DBP (k4 vs k5) [22], socioeconomic status, demographic discrepancies, daily intake of salt, and physical activity levels.

Many studies suggested that the prevalence of childhood obesity in China is on the rise. Dong et al. reported that prevalence of overweight, obesity in children aged 12–14 years old was 11.9 and 7.1% respectively in 2011, in Shandong, China [32]. Another cross-sectional survey found the prevalence of overweight, obesity among children aged 13–15 years was 14.65 and 10.61% for boys, 9.75 and 5.85% for girls [33]. In Guangzhou, a 4 years’ cohort study from 2007 to 2011 among children indicated the prevalence of overweight and obesity was 7.7%, 6.3% in 2007, and 12.4, 8.3% in 2011, respectively [34]. In Shanghai [35], the schoolchildren overweight and obesity prevalence was 11.27, and 13.53% in 2009, respectively, and 12.5, 15.7% in Tianjin, 2011 [36]. A cross-sectional study conducted in Beijing in 2004 showed that overweight or obesity prevalence in those aged 13–15 years was 11.7, 8.7%, respectively [37]. Consistent with previous studies, we also found that prevalence of overweight, obesity in 2639 junior school students (1218 boys and 1421 girls), aged 12–15 years old was 17.9 and 8.1%, respectively. The present study showed that boys were nearly twice as likely to be overweight or obese than girls. However, 32.2% of children in the United States aged 2 to 19 years were overweight and 17.3% were obese in 2011 to 2012 [38], which were higher than that in our study. All of these studies showed that overweight and obesity has gender disparities. The reason may be due to demographic characteristics, socio-economic status, diet habits and physical activities. Compared with normal weight students, those who were overweight or obese showed a significantly higher prevalence of hypertension (see Table 3), i.e. overweight and obesity are strongly associated with hypertension among adolescents, consistent with previous reports [39, 40].

Because of the smart phone, we can not only watch video programs, but also real-time communications, interactive games, online shopping and so on. Therefore, not only will it lead to the related problems brought about by watching TV in the past, but also the problems caused by unhealthy use of mobile phones are more extensive and complex. In recent years, many studies have found that problematic smartphone use is associated with some health hazards, ranging from psychosocial disorders such as anxiety, depression and sleep problems [24, 41–44] to potentially fatal injuries caused by traffic accidents [45].

The prevalence of smartphone addiction among our participants were 22.8% (22.3% for females and 23.2% for males), lower than the results of the previous study conducted in 1441 undergraduate students in China [45] (29.8%, 30.3% in males and 29.3% in females), measured with the same scale tool (SAS-SV). While the prevalence of smartphone addiction in 1519 students from 127 Swiss vocational school classes was 16.9% [25]. The prevalence of smartphone addiction in junior high school students in South Korea was 24.8% [24], while the prevalence of smartphone addiction was 12.5% for Spanish and 21.5% for francophone Belgians, respectively [46]. These discrepancies could be due to the differences among the participants. Another results using other scales also indicate that smartphone addiction is becoming a public health problem that cannot be ignored [17, 47, 48].

Interestingly, hypertensive adolescents also have a higher rate of smartphone addiction. Among 428 hypertensive adolescents, 61.7% were addicted to smartphones, compared with 15.3% of those without hypertension (338/2211). This suggests that adolescents with hypertension are more likely to be attracted to smartphones, but the exact mechanism still needs further study.

We found that 48.6% of 518 adolescents with poor sleep quality had hypertension, much higher than those with good sleep quality (8.3%). Previous studies had shown that sleep deprivation was associated with high blood pressure [49–51]. Poor quality sleep may be at higher risk of elevated blood pressure that could lead to cardiovascular disease in adulthood [51]. A number of studies had shown that addiction to mobile phones was associated with sleep problems in adolescents [41, 43, 52, 53]. Our results also showed that there was a similar relationship between smartphone addiction and sleep quality. The risk of hypertension was higher among those with smartphone addiction (OR = 2.205, 95% CI:1.273–3.820) and poor sleep quality (OR = 4.243, 95% CI:2.429–7.411). Studies have suggested that screen media exposure leads to obesity in children and adolescents [54]. We speculate that obesity and poor sleep quality may be underlying mechanisms by which smartphone addiction, as a screen media, affects blood pressure levels in adolescents.

One limitation of this study was that BP levels were obtained by a single visit. Therefore, the prevalence of hypertension was likely overestimated in the current study. Nevertheless, the BP measurement was performed at school, in the adolescents’ usual environment, which would have helped reduce any ‘white-coat’ effect. The second limitation was that cross-sectional nature of the study limits drawing of inferences about causation. This can be resolved by using longitudinal studies to further determine the causation. The last but not least limitation was that we did not assess other factors associated with high blood pressure, such as daily eating habits (especially salt intake), physical activity, genetic factors, usage of stimulants for treating attention deficit hyperactivity disorder which is associated with hypertension in adolescents [55] and depression [56] that may affect our findings.

## Conclusions

This study demonstrated that, among Chinese junior school students aged 12–15 years, the prevalence of elevated blood pressure was high, and associated with the increasing prevalence of overweight/obesity, poor sleep quality and smartphone addiction, respectively. These results suggest that smartphone addiction may be a new risk factor for high blood pressure in adolescents.

## Data Availability

The datasets used and/or analyzed during the current study are available from the corresponding author on reasonable request.

## Abbreviations

BMI: Body mass index
BP: Blood pressure
DBP: Diastolic blood pressure
HTN: Hypertension
SBP: Systolic blood pressure
